# Development of a new health-related quality of life measure for people with diabetes who experience hypoglycaemia: the Hypo-RESOLVE QoL

**DOI:** 10.1007/s00125-024-06182-9

**Published:** 2024-05-22

**Authors:** Jill Carlton, Philip A. Powell, Melanie Broadley, Bastiaan E. de Galan, Simon Heller, Jonathan Comins, Myriam Rosilio, Frans Pouwer, Mari-Anne Gall, Christopher J. Child, Rory J. McCrimmon, Donna Rowen

**Affiliations:** 1 Sheffield Centre of Health and Related Research (SCHARR), University of Sheffield, Sheffield, UK; 2 Department of Psychology, University of Southern Denmark, Odense, Denmark; 3 Department of Medicine, Radboud University Medical Centre, Nijmegen, the Netherlands; 4 Department of Internal Medicine, Maastricht University Medical Center+, Maastricht, the Netherlands; 5 CARIM School for Cardiovascular Disease, Maastricht University, Maastricht, the Netherlands; 6 Department of Oncology and Metabolism, University of Sheffield, Sheffield, UK; 7 Medical Science Innovation, Centre of Expertise, Patient Focused Drug Development, Novo Nordisk A/S, Søborg, Denmark; 8 Diabetes & Obesity Medical Unit, Eli Lilly & Company, Neuilly sur seine, France; 9 Steno Diabetes Center Odense, Odense, Denmark; 10 Medical & Science, Diabetes, Clinical Drug Development, Novo Nordisk A/S, Søborg, Denmark; 11 Eli Lilly and Company, Lilly Diabetes and Obesity, Bracknell, UK; 12 Systems Medicine, School of Medicine, University of Dundee, Dundee, Scotland

**Keywords:** Diabetes, Health-related quality of life, Hypoglycaemia, Patient-reported outcome measure, Preference-based measure, Psychometrics, Qualitative research

## Abstract

**Aims/hypothesis:**

Valid and reliable patient-reported outcome measures are vital for assessing disease impact, responsiveness to healthcare and the cost-effectiveness of interventions. A recent review has questioned the ability of existing measures to assess hypoglycaemia-related impacts on health-related quality of life for people with diabetes. This mixed-methods project was designed to produce a novel health-related quality of life patient-reported outcome measure in hypoglycaemia: the Hypo-RESOLVE QoL.

**Methods:**

Three studies were conducted with people with diabetes who experience hypoglycaemia. In Stage 1, a comprehensive health-related quality of life framework for hypoglycaemia was elicited from semi-structured interviews (*N*=31). In Stage 2, the content validity and acceptability of draft measure content were tested via three waves of cognitive debriefing interviews (*N*=70 people with diabetes; *N*=14 clinicians). In Stage 3, revised measure content was administered alongside existing generic and diabetes-related measures in a large cross-sectional observational survey to assess psychometric performance (*N*=1246). The final measure was developed using multiple evidence sources, incorporating stakeholder engagement.

**Results:**

A novel conceptual model of hypoglycaemia-related health-related quality of life was generated, featuring 19 themes, organised by physical, social and psychological aspects. From a draft version of 76 items, a final 14-item measure was produced with satisfactory structural (χ^2^=472.27, *df*=74, *p*<0.001; comparative fit index =0.943; root mean square error of approximation =0.069) and convergent validity with related constructs (*r*=0.46–0.59), internal consistency (α=0.91) and test–retest reliability (intraclass correlation coefficient =0.87).

**Conclusions/interpretation:**

The Hypo-RESOLVE QoL is a rigorously developed patient-reported outcome measure assessing the health-related quality of life impacts of hypoglycaemia. The Hypo-RESOLVE QoL has demonstrable validity and reliability and has value for use in clinical decision-making and as a clinical trial endpoint.

**Data availability:**

All data generated or analysed during this study are included in the published article and its online supplementary files (https://doi.org/10.15131/shef.data.23295284.v2).

**Graphical Abstract:**

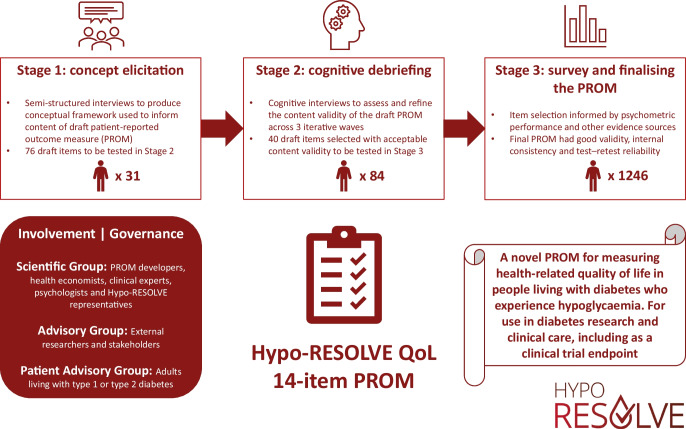

**Supplementary Information:**

The online version of this article (10.1007/s00125-024-06182-9) contains peer-reviewed but unedited supplementary material.


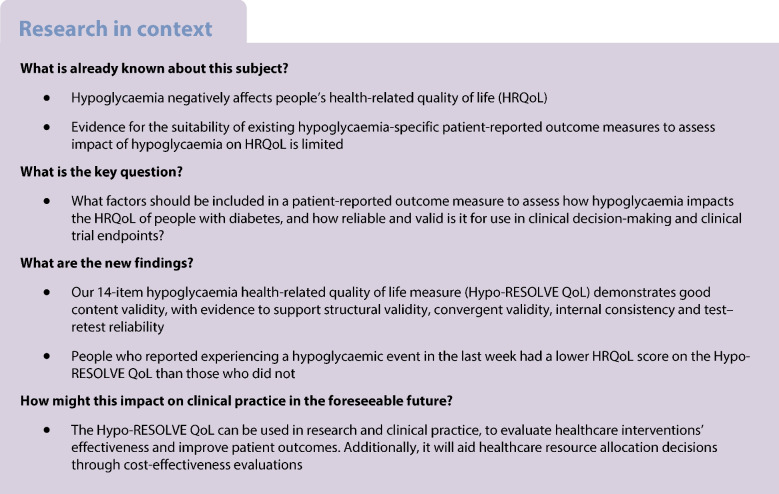


## Introduction

Hypoglycaemia impacts the health and wellbeing of people with diabetes [1–3], which can be subjectively quantified using patient-reported outcome measures (PROMs). Valid and reliable PROMs are vital for accurately assessing real-life health impacts and the effectiveness of care for people with diabetes. PROMs can be developed further to evaluate the cost-effectiveness of healthcare interventions (i.e. preference-based scoring methods). A recent review has questioned whether existing hypoglycaemia-specific PROMs can capture the full impact of hypoglycaemia on health-related quality of life (HRQoL) [4]. HRQoL is increasingly recognised as an important outcome in healthcare settings and in clinical trials [5].

Carlton et al highlighted gaps in evidence for psychometric performance, especially among legacy measures [4]. In particular, there was insufficient supporting evidence for the content and structural validity of the reviewed PROMs to assess the quality of life (QoL) impacts of hypoglycaemia, raising questions over their suitability for use for this purpose. While a recent hypoglycaemia-specific PROM assessing the impact on QoL has been published [6], this was a rapid adaptation of an existing diabetes-specific measure. As acknowledged by the authors, this measure did not follow full developmental rigour (e.g. supporting qualitative content validity interviews [7]) and was not developed for use in health economic evaluations or cost-effectiveness analysis as a result of clinical trials [6].

The aim of this mixed-methods, multi-stage project was to develop an HRQoL PROM for use in hypoglycaemia that had demonstrable validity and reliability and enabled patient experiences to be incorporated into clinical shared decision-making and as a clinical trial endpoint. The terms HRQoL and QoL are often used interchangeably and/or not well-defined. We define HRQoL as ‘a multidimensional concept that includes the physical, psychological and social functioning associated with an illness or its treatment’ [8]. An additional aim was for the PROM to be amenable to adaptation for use in cost-effectiveness analyses of new healthcare technologies for hypoglycaemia (via preference-based scoring) [9]. The need for this PROM was endorsed by an international collaboration of clinicians, scientists, industry partners and people with diabetes via the Hypoglycaemia REdefining SOLutions for better liVEs (Hypo-RESOLVE) project [10].

**Fig. 1:**
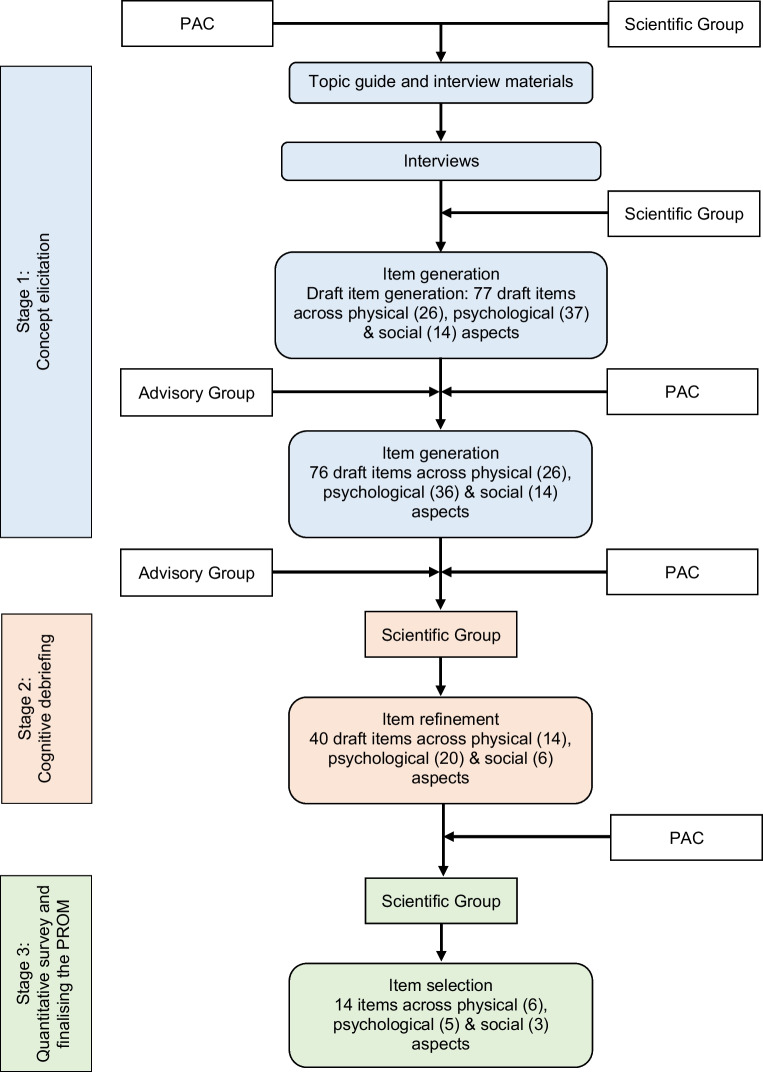
Overview of Hypo-RESOLVE QoL development

The hypoglycaemia health-related QoL measure (Hypo-RESOLVE QoL) was developed following best practice (Fig. 1) [7, 11], involving a series of studies with people with diabetes. This included qualitative work eliciting a comprehensive understanding of hypoglycaemia-related HRQoL impacts on people with diabetes, further qualitative work refining the draft PROM and an assessment of its psychometric performance in a large sample of individuals with diabetes. The work had three governance groups involved at key stages, including: an Advisory Group of researchers and stakeholders external to the Hypo-RESOLVE Consortium; a patient advisory committee (PAC) comprising adults living with type 1 or type 2 diabetes and representatives from the IDF and JDRF drawn from Hypo-RESOLVE patient advisors (https://hypo-resolve.eu/network/advisors); and a Scientific Group comprising PROM developers, health economists, clinical experts and stakeholder representatives from the wider Hypo-RESOLVE Consortium.

## Methods

The development of the Hypo-RESOLVE QoL is summarised in Fig. 1 and an associated protocol [12]. We used mixed-methods, as follows: concept elicitation interviews (to generate an HRQoL framework and draft PROM content); cognitive debriefing interviews (to validate and refine content); and a large quantitative survey (to assess psychometric performance). The final version of the Hypo-RESOLVE QoL was informed by psychometric analyses and complementary evidence, including a translatability assessment and stakeholder engagement. Ethics approval was obtained from the UK National Health Service (NHS; REC reference: 20/NI/0048) and all participants gave informed consent.

### Stage 1: concept elicitation

Items (questions) for the draft PROM were informed by an HRQoL framework elicited through semi-structured interviews with individuals with diabetes who reported at least one self-defined hypoglycaemic event (‘hypo’) in the previous 12 months. Adult participants (≥18 years) were recruited from a large NHS site, purposively sampled across age, sex and type and duration of diabetes. Demographic data were taken from hospital records. Recruitment continued until data saturation (defined by no new codes emerging for three interviews [13]) in a sample of sufficient breadth. Interviews were conducted online or by telephone due to COVID-19. A topic guide, informed by a previous review [4] and developed with the PAC, was produced to cover aspects of HRQoL potentially relevant to hypoglycaemia (see our online supplementary files, hosted by University of Sheffield; 10.15131/shef.data.23295284.v2, supplementary file A). Interviews with 31 people with diabetes were conducted between September 2020 and April 2021. The mean duration of each interview was 42 min (range 20–77 min). Participant demographics are shown in Table 1.

**Table 1:** Participant demographics

Demographic	*n*
Stage 1 (*N*=31)	
Age	
18–30 years	9
31–65 years	15
66+ years	7
Sex at birth	
Male	16
Female	15
Diabetes type	
Type 1	21
Type 2	10
Diabetes duration	
0–5 years	9
6–10 years	5
11–20 years	7
21+ years	10
Stage 2 (*N*=70)	
Age	
18–30 years	16
31–65 years	39
66+ years	15
Sex at birth	
Male	32
Female	38
Diabetes type	
Type 1	50
Type 2	19
Other	1
Diabetes duration	
0–5 years	17
6–10 years	9
11–20 years	20
21+ years	24
Stage 3 (*N*=1246)	
Age (mean ± SD 48.87 ± 16.29 years, range 18–91 years)	
Prefer not to say/missing^a^	1
Sex at birth	
Female	688
Male	557
Prefer not to say/missing	1
Gender	
Woman (including transgender woman)	684
Man (including transgender man)	558
Non-binary/gender-fluid	4
Ethnicity	
Asian or Asian British	25
Black, Black British, Caribbean or African	9
Mixed or multiple ethnic groups	26
Other ethnic group	5
White	1175
Prefer not to say/missing	6
English as a first language	
No	53
Yes	1193
Education	
GCSE or equivalent secondary school qualification	241
A-level or equivalent post-secondary level qualification	292
Bachelor’s or equivalent first degree	388
Master’s or equivalent higher degree	183
PhD or equivalent doctoral qualification	44
None of the above	77
Prefer not to say/missing	21
Employment	
Employed	797
Retired	271
Student/in training	35
Unemployed (due to disability or sickness)	87
Unemployed (job-seeking)	17
Unemployed (not seeking work)	27
Other^b^	3
Prefer not to say/missing	9
Diabetes type	
Type 1 diabetes	993
Type 2 diabetes	213
Gestational diabetes	13
Other	26
Prefer not to say/missing	1
Diabetes duration (mean ± SD 22.68 ± 15.70 years, range 0–70 years)	
Prefer not to say/missing^c^	7
Medication^d^^,e^	
Oral glucose-lowering medication	226
Insulin	1156
Non-insulin injectable medication	43
None of the above	16
Insulin type^e,f^	
Background or long-acting insulin only	50
Insulin pump therapy (continuous subcutaneous insulin infusion)	304
Mixed insulin	148
Multiple daily insulin injections	647
Prefer not to say/missing	6
Insulin duration^c,f^ (mean ± SD 22.40 ± 16.23 years, range 0–70 years)	
Prefer not to say/missing^c^	7
Glucose monitoring^d^	
Finger prick blood glucose monitor	549
Freestyle Libre	137
Freestyle Libre-2	605
Other continuous glucose monitor	186
Urine monitor	4
Do not monitor	21
Other^g^	2
Prefer not to say/missing	3
During the last month, how often did you deliberately run your blood glucose ‘high’ to avoid having a hypo (or ‘going low’)?	
Never	409
Rarely	296
Sometimes	404
Often	112
Almost always	23
Prefer not to say/missing	2
Comorbid conditions^d^	
Heart disease	100
Kidney problems	85
Liver problems	30
Other endocrine diseases	229
Peripheral neuropathy	161
None of the above	795
Prefer not to say/missing	5
Gold Score	
Mean ± SD	2.54 (1.61)
Range	1–7
Prefer not to say/missing	4

^a^One response was missing but the participant consented to the conditions of participation and was inferred as being over 18 years of age because of the length of time they reported living with diabetes

^b^Twenty-three ‘other’ free-text responses were recoded into the employment categories, the remaining three responses covered informal caregiving

^c^Where age (diabetes duration OR insulin duration) was <0 years, then values on these variables were recoded as missing

^d^Categories are not mutually exclusive

^e^Free-text responses provided were recoded using input from clinicians

^f^Only includes people who are on insulin

^g^Fourteen ‘other’ free-text responses were recoded into glucose monitoring categories, the remaining two responses covered Freestyle Libre (version unknown)

A-level, Advanced level (typically taken at age 18 years in the UK); GCSE, General Certificate of Secondary Education (typically taken at age 16 years in the UK); PhD, Doctor of Philosophy; Gold Score, measure of hypoglycaemia awareness

Interviews were conducted by two senior HRQoL researchers with qualitative experience. Interviews were audio recorded, transcribed verbatim and anonymised. Data was coded iteratively alongside data collection using Framework Analysis [14], following Gale et al [15]. Transcripts were analysed independently, with both researchers analysing their own interviews and dual coding 50%. An initial codebook informed by the draft HRQoL framework was used and refined based on emerging themes. Groups of four transcripts were coded at any one time, before the researchers met to discuss their coding and revise the working framework.

A final thematic framework, developed after all transcripts were coded, was used to inform draft PROM content in consultation with the PAC and Scientific Group. Where appropriate, multiple potential items were included to be tested in Stage 2. Item development followed a set of rules developed in previous research [16].

### Stage 2: cognitive debriefing

Cognitive debriefing interviews were conducted with individuals with diabetes and clinicians to assess and refine the content validity of the draft PROM and reduce redundancy. Interviews were conducted online or by telephone in three iterative waves and facilitated by a topic guide (10.15131/shef.data.23295284.v2, supplementary file B).

Adult participants (≥18 years) were recruited from a large NHS site in the UK and from the Diabetologist’s Office, Diabetes Association, social media and word of mouth in Germany. Participants were purposively sampled across age, sex, type and duration of diabetes. Demographic data was taken from hospital records. Participants received the draft items in advance. Relevance (are the items, response options and recall period appropriate?), comprehensiveness (are all key concepts included?) and comprehensibility (is the PROM content understood as intended?) were assessed [7, 17]. Where items covered overlapping constructs (e.g. ‘I felt sad’ and ‘I felt depressed’), participants’ preference was elicited. Items were split into three aspects of HRQoL (physical, social, psychological), mirroring the Stage 1 framework. Cognisant of potential response burden, each participant was asked to review up to 40 items. Interviews were recorded and transcribed verbatim. Detailed notes and transcripts were used to refine draft PROM content. Eighty-four interviews were conducted (70 people with diabetes and 14 clinicians) between August 2021 and March 2022 across three waves. Participant demographics are shown in Table 1.

### Stage 3: quantitative survey and finalising the PROM

The draft PROM content was administered as part of a survey to assess psychometric performance. The survey was completed by a large UK sample of people with diabetes (≥18 years) who self-reported at least one hypoglycaemic episode in the past 12 months, recruited from 25 NHS sites in the UK (10.15131/shef.data.23295284.v2, supplementary file C). Sampling involved a mixture of targeted and convenience strategies, including using existing patient databases to distribute e-mails and letters, putting up posters and giving invitations in clinic. To ensure sufficient sampling breadth for the psychometric analyses, a minimum sample of 1000 participants was targeted (at least 200 with type 2 diabetes).

The draft Hypo-RESOLVE QoL was presented alongside sociodemographic questions including self-reported gender, clinical background measures and other measures of HRQoL in a fixed order. The following measures were included: the Gold Score [18] and hypoglycaemia awareness questionnaire (HypoA-Q) [19] measures of hypoglycaemia awareness; a 100-point visual analogue scale (VAS) of hypoglycaemia-related QoL (‘at the moment’ and ‘past 4-weeks’ versions); DAWN2 Impact of Diabetes Profile (DIDP) [20] (‘currently’ and ‘past 4-weeks’ versions); and a generic measure of health-related QoL (EQ-5D-5L) [21]. A copy of the survey, with further details on measures used, is available (10.15131/shef.data.23295284.v2, supplementary file D).

The survey was hosted online, with paper surveys provided upon request. To assess test–retest reliability of the Hypo-RESOLVE QoL, a convenience sample, from two NHS sites, of online participants who expressed an interest were invited to take part again, a minimum of 4 weeks later. Survey responses at these sites were linked to participants’ clinical data obtained from medical records (HbA_1c_ and continuous glucose monitoring data, where available) but these data were not used in the development of the PROM.

Survey data were cleaned and subject to a priori defined quality checks [12]. Psychometric analyses were conducted in R (v4.2.2; https://cran.r-project.org/bin/windows/base/old/4.2.2/) iteratively, using techniques described by Dima [22]. A sample of 1246 participants was used for analysis. Participant demographics are shown in Table 1. Hypo-RESOLVE QoL data was summarised descriptively, including the distribution of responses (to explore potential ceiling and/or floor effects) and missing data. Classical test theory (i.e. correlations, confirmatory factor analysis with maximum likelihood estimation [one-factor and three-factor models], Cronbach’s α) and item response theory (IRT; i.e. Mokken scale analysis based on a monotone homogeneity model; IRT partial credit model; differential item functioning [DIF]) analyses of the draft Hypo-RESOLVE QoL were undertaken to help inform final item selection. A partial credit model showed superior fit to a rating scale model in a likelihood ratio test. DIF was assessed by diabetes type (type 1 vs type 2) and gender (woman vs man) using logistic regression with IRT θ estimates as the conditioning variable.

A priori criteria used for assessing psychometric performance were developed and used to interpret results (Table 2). Tests that necessitated item grouping were based on the underlying theoretical HRQoL model (i.e. physical, social, psychological). Prior to parametric IRT analyses, sufficient unidimensionality was assessed using Mokken scale analysis homogeneity coefficients of the subscales. Mokken scale analysis was also used to assess assumptions of local dependence and monotonicity. Towards the latter stages of item selection (*n*=18 items), the same tests were also conducted on the overall scale as a total HRQoL score, which was assessed for sufficient homogeneity.

**Table 2:** Psychometric analyses used to help inform item selection (Stage 3)

Analysis	Benchmarking criteria used to determine acceptable performance
Descriptives	
Floor and ceiling effects	>5% and <40% of responses in the floor/ceiling response category
Non-applicable/missing responses	<20% of responses are non-applicable, <5% of responses are missing (paper surveys only)
Mokken scale analysis^a^	
Coefficients of homogeneity (H)	H≥0.30 [34]
Local independence (conditional association)	Item does not significantly violate local independence assumption [34]
Monotonicity	Item does not significantly violate monotonicity (critical value ≥80) [35]
IRT partial credit model^b^	
Item fit (infit and outfit)	Mean squares between 0.6 and 1.4 [36]
Item category thresholds	Item category threshold parameters are ordered [37]
Differential item functioning (DIF)^c^	Non-significant DIF (α<0.01) in logistic regression with IRT θ estimates as the conditioning variable, and McFadden *R*^2^<0.02 [38]
Classical test theory	
Confirmatory factor analysis, with a one-factor baseline and three-factor hypothesised model (physical, psychological, social)^d^	CFI ≥0.9 [29]; RMSEA <0.08 [39]
Cronbach’s α^e^	Cronbach’s α ≥0.7 [40]
Item correlations	
Polychoric intercorrelations between items^e^	Intercorrelations between items <0.7, indicating non-redundancy [41]
Spearman correlations between items and the hypoglycaemia-related QoL VAS (4 weeks)^e^	Non-trivial correlation (*r*_s_≥0.2) [42]
Spearman correlations between items and HypoA-Q question 1 (i.e. number of hypos in the previous week)^e^	Non-trivial correlation (*r*_s_≥0.2) [42]

R package dplyr was used to help clean and recode data prior to analysis

^a^Psychometric analyses were conducted using R package mokken

^b^Psychometric analyses were conducted using R package eRm

^c^Psychometric analyses were conducted using R package lordif

^d^Psychometric analyses were conducted using R package lavaan

^e^Psychometric analyses were conducted using R package psych

Following final item selection (Stage 2.3), the Hypo-RESOLVE QoL was scored summatively, and Pearson correlations were estimated. This facilitated an initial analysis of the construct validity of the Hypo-RESOLVE QoL based on a priori hypotheses about its associations with other measures [12]. We expected to demonstrate convergent validity by the Hypo-RESOLVE QoL correlating moderately (at *r*≥0.3) with the VAS, DIDP and EQ-5D-5L, with the size of coefficient larger for more similar constructs (i.e. VAS > DIDP > EQ-5D-5L).

Test–retest reliability for the Hypo-RESOLVE QoL was estimated with the intraclass correlation coefficient (ICC) on a subset of repeat participants who demonstrated sufficient stability in the construct of interest (whose differences in scores on the VAS were within 1 SD of the mean). A two-way random effects model with absolute agreement and single-unit parameters was estimated. ICC values suggest reliability as follows: 0.5–0.75 moderate reliability; 0.75–0.9 good reliability; and >0.9 excellent reliability [23].

In addition to psychometrics, to produce the final version of the Hypo-RESOLVE QoL multiple sources of evidence were considered. This included: Stage 2 findings; a translatability assessment of draft PROM content in eight languages selected by the Hypo-RESOLVE Consortium (10.15131/shef.data.23295284.v2, supplementary file E); published criteria for selecting items for use in health economics [16]; and PAC and expert Advisory Group consultations. To facilitate decision-making, a traffic-light system was used to indicate whether responses to each item (from each source) were positive (green), mixed (amber) or negative (red) (10.15131/shef.data.23295284.v2, supplementary file E) [24, 25]. Decisions were made iteratively (allowing for updates to the psychometric information when changes were made) to help define the final PROM.

## Results

### Stage 1: item generation

Three higher-level themes (physical, social, psychological) were used to organise the qualitative data, consisting of 19 subthemes (10.15131/shef.data.23295284.v2, supplementary file F), from which items were generated. The full list of draft items (*n*=76) and how they mapped onto underlying themes is available (10.15131/shef.data.23295284.v2, supplementary file G). Items were rephrased to allow subsequent testing of severity and frequency response options. Five-item response scales and a 7 day recall period were initially selected for testing at Stage 2.

### Stage 2: initial item testing

Reductions in the draft items were made in each wave, due to perceived redundancy or overlapping items (10.15131/shef.data.23295284.v2, supplementary file H). In the first two waves, participants provided suggestions for rewording of some items (e.g. ‘I could do what I wanted to do [in my life]’) and identified new items (e.g. ‘I had interrupted sex’). A preference for frequency-based response options was determined after Wave 2. Participants favoured the use of the five-point response scale. Most participants preferred a longer recall period than 7 days, so a 1 month recall period was tested at Wave 2 and approved by participants (rephrased as ‘4 weeks’ for consistency across months). Minor modifications were made to the instructions following Waves 1 and 2 to improve clarity (e.g. to emphasise that participants should consider the overall impact of hypoglycaemia). At the end of Wave 2, decisions on the final draft PROM content were made and tested in Wave 3. Six items were dropped in Wave 3 and no further substantive problems with the draft PROM were identified. All items were understood as intended and considered relevant to HRQoL in hypoglycaemia. The draft PROM was deemed sufficiently comprehensive and a revised draft 40-item version was agreed (10.15131/shef.data.23295284.v2, supplementary file I).

### Stage 3: final item selection

The PAC rated two items and the Hypo-RESOLVE Advisory Group nine items as potentially redundant (10.15131/shef.data.23295284.v2, supplementary file E). The translatability assessment identified two problematic items (‘I felt ashamed’ and ‘my sex life was negatively affected’) if the content of the measure was adapted for use in other countries and/or cultures.

The online survey was accessed 2562 times and 213 paper surveys were returned. Of these, 821 online accesses did not result in participation, 307 responses were incomplete (280 left the survey prior to the Hypo-RESOLVE QoL and 27 started but did not complete the Hypo-RESOLVE QoL), 40 responses were duplicates and 321 responses were screened out (did not meet the inclusion criteria; including 18 paper surveys from which data were not inputted). To ensure data quality, 19 responses were excluded for being quicker than the estimated survey reading speed (at 300 words per minute [26]) or taking longer than 24 h, and 21 responses were excluded for straight-lining >25% of the Hypo-RESOLVE QoL, including opposing items (psychometric results are also presented with these participants included, 10.15131/shef.data.23295284.v2, supplementary file E). Seventy-five responses were examined for potential multivariate outliers but no suspicious or implausible response patterns were observed. This resulted in a sample of 1246 for analysis. A breakdown of responses per recruiting site is available (10.15131/shef.data.23295284.v2, supplementary file C).

Clinical and sociodemographic characteristics of the sample are given in Table 1. Mean age was 48.87 (SD 16.29) years, with a slight majority of women (*n*=684, 54.9%). Almost all participants were White (*n*=1175, 94.3%), with the majority having type 1 diabetes (*n*=993, 79.7%). The mean self-reported duration of diabetes was 22.68 (SD=15.70) years.

An initial analysis of the 40-item Hypo-RESOLVE QoL showed an unacceptable fit to the tripartite HRQoL model (with physical, social and psychological factors): χ^2^=5624.42, *df*=737, *p*<0.001; CFI=0.754; RMSEA=0.087 (90% CI 0.085, 0.090), *p*<0.001. Several potential problems with items were identified in the floor/ceiling, Mokken and IRT analysis. Several high intercorrelations between items were observed, particularly in the psychological domain, suggesting potential redundancy. A full set of psychometric results is available (10.15131/shef.data.23295284.v2, supplementary file E).

Eleven items were dropped from the Hypo-RESOLVE QoL. These included items that substantially overlapped with other items (i.e. intercorrelations ≥0.7) and some of the worst psychometric performers (unless there was a strong theoretical rationale for keeping the item). All decisions on which items were kept and dropped and the rationale are available in an item-tracking matrix (10.15131/shef.data.23295284.v2, supplementary file H).

The 29-item revised Hypo-RESOLVE QoL displayed an improved fit but this was still below acceptable levels: χ^2^=2482.54, *df*=374, *p*<0.001; CFI=0.840; RMSEA=0.079 (90% CI 0.076, 0.082), *p*<0.001. Notable problems also remained at the item level. A further 11 items were dropped from the scale, which showed a range of violations in the Mokken and/or IRT analysis (10.15131/shef.data.23295284.v2, supplementary file J).

The 18-item version of the measure revealed an acceptable fit to the HRQoL model: χ^2^=966.69, *df*=132, *p*<0.001; CFI=0.910; RMSEA=0.075 (90% CI 0.070, 0.079), *p*<0.001. Fewer potential problems were evident at the item level. However, some problems remained, including violations of local independence, potential floor effects and problematic DIF by diabetes type. At this point, an increasing trade-off was being made between item coverage (comprehensiveness) and statistical performance. In consultation with the Scientific Group and PAC, four final items were dropped from the scale (10.15131/shef.data.23295284.v2, supplementary file K).

The final, 14-item Hypo-RESOLVE QoL demonstrated an acceptable fit to the three-factor HRQoL model: χ^2^=472.27, *df*=74, *p*<0.001; CFI=0.943; RMSEA=0.069 (90% CI 0.063, 0.075), *p*<0.001. This showed a superior fit to a one-factor model: χ^2^=836.41, *df*=77, *p*<0.001; CFI=0.891; RMSEA=0.093 (90% CI 0.088, 0.099), *p*<0.001. Potential psychometric problems were minimised but some minor statistical issues remained (10.15131/shef.data.23295284.v2, supplementary file L). Three items violated local independence at the domain level, although not when considered as part of the overall scale. One item at the domain level and two items at the overall scale level showed disordered thresholds. However, this was a small proportion of the total items, so the original response options were retained. One item showed a potential clustering of responses at the floor (54.2%) and another item was just below the threshold for good IRT item fit at the domain level and showed potential non-uniform DIF for diabetes type on the overall scale (McFadden *R*^2^ 0.030; see electronic supplementary material [ESM] Fig. 1). It was deemed important to retain both to retain to facilitate the calculation of a social HRQoL domain score (which requires three or more items). All items were endorsed by at least 50% of participants in the cognitive debriefing study and PAC consultation and had a positive translatability assessment. Ultimately, no further revisions were considered justified.

### Scoring and relationship with other variables

Simple summative scores were calculated for the three subdomains and total score of the Hypo-RESOLVE QoL so that a higher score represented better HRQoL (4 = none of the time, 0 = most or all of the time; the item ‘I could do what I wanted to do in my life’ is reverse-scored). Cronbach’s α for the Hypo-RESOLVE QoL total score was 0.91, with values of 0.85, 0.78 and 0.81 for the physical, social and psychological subscales, respectively. Scores for the HRQoL measures included in the study are in shown in Table 3.

**Table 3:** HRQoL measures used in Stage 3

Measure	Mean ± SD	Range	Prefer not to say/missing (*n*)
Hypoglycaemia-related QoL VAS (at the moment)	70.54 ± 21.48	0–100	2
Hypoglycaemia-related QoL VAS (past 4 weeks)	70.65 ± 21.65	0–100	2
HypoA-Q (impaired awareness subscale)	6.07 ± 3.93	0–20	167^a^
DIDP (current)	4.74 ± 0.85	1–7	7
DIDP (past 4 weeks)	4.71 ± 0.84	1–7	21
EQ-5D-5L	0.725 ± 0.282	−0.454 to 0.989	32
Hypo-RESOLVE QoL (physical subscale)	11.33 ± 4.48	0–20	8
Hypo-RESOLVE QoL (social subscale)	8.55 ± 2.71	0–12	2
Hypo-RESOLVE QoL (psychological subscale)	14.97 ± 5.16	0–24	105^b^
Hypo-RESOLVE QoL (total score)	34.69 ± 10.89	0–56	114^b^

^a^An initial error on the online version of the HypoA-Q was identified early on in data collection where the incorrect (previous) response option labels were included on three items. This affected 12% (*n*=154) of responses, which were recoded as missing

^b^Items 38–40 on the Hypo-RESOLVE QoL permitted an ‘NA’ response

The Hypo-RESOLVE QoL total score did not significantly differ between people with type 1 diabetes or type 2 diabetes (mean ± SD 34.56 ± 10.54 vs 35.31 ± 12.64, respectively; *t*=−0.73, *df*=217.46, *p*=0.464, *d*=−0.07). Men reported a higher total HRQoL than women (mean ± SD 36.38 ± 11.10 vs 33.44 ± 10.53, respectively; *t*=−4.52, *df*=1038.1, *p*<0.001, *d*=0.27). The Hypo-RESOLVE QoL total score was significantly greater in those who did not report having a hypoglycaemic event in the last week (*n*=224) than those who did (*n*=1022) (mean ± SD 39.6 ± 10.78 vs SD 33.75 ± 10.66, respectively; t=6.73, *df*=255.32, *p*<0.001, *d*=0.55), suggesting provisional known-group validity.

Correlations (Pearson and point-biserial) between the HRQoL measures and background characteristics are shown in Table 4. As hypothesised [12], moderate-to-large correlations were observed between the Hypo-RESOLVE QoL total score and the VAS (4 weeks) (*r*=0.59), DIDP (*r*=−0.55) and the EQ-5D-5L (*r*=0.46), in the expected order of magnitude. Thus, the measure showed preliminary convergent validity.

**Table 4:** Pearson and point-biserial correlations between HRQoL measures and background characteristics (Stage 3)

Measure	(1) Hypo-RESOLVE QoL total	(2) Hypo-RESOLVE QoL physical	(3) Hypo-RESOLVE QoL social	(4) Hypo-RESOLVE QoL psychological	(5) Hypoglycaemia QoL VAS (4 weeks)	(6) DIDP (4 weeks)	(7) HypoA-Q impaired awareness	(8) EQ-5D-5L
(1) Hypo-RESOLVE QoL total	–	0.89	0.83	0.92	0.59	−0.55	−0.23	0.46
(2) Hypo-RESOLVE QoL physical	0.89	–	0.64	0.70	0.52	−0.47	−0.17	0.34
(3) Hypo-RESOLVE QoL social	0.83	0.64	–	0.69	0.49	−0.46	−0.18	0.32
(4) Hypo-RESOLVE QoL psychological	0.92	0.70	0.69	–	0.56	−0.53	−0.23	0.49
(5) Hypoglycaemia QoL VAS (4 weeks)	0.59	0.52	0.49	0.56	–	−0.42	−0.18	0.39
(6) DIDP (4 weeks)	−0.55	−0.47	−0.46	−0.53	−0.42	–	0.07	−0.35
(7) HypoA-Q impaired awareness	−0.23	−0.17	−0.18	−0.23	−0.18	0.07	–	−0.17
(8) EQ-5D-5L	0.46	0.34	0.32	0.49	0.39	−0.35	−0.17	–
(9) HypoA-Q / no. of hypoglycaemia events (past week)	−0.22	−0.21	−0.24	−0.18	−0.21	0.18	0.13	−0.01
(10) Age (years)	0.27	0.28	0.25	0.21	0.13	−0.14	0.10	−0.11
(11) Identifies as a woman	−0.13	−0.15	−0.06	−0.12	−0.04	0.06	−0.01	−0.05
(12) Non-White ethnicity	−0.02	−0.02	−0.03	−0.02	−0.07	0.01	0.00	−0.03
(13) Has English as a first language	−0.01	−0.02	−0.02	0.01	−0.01	0.01	0.03	−0.02
(14) Has a degree	0.03	0.01	0.01	0.05	0.03	0.06	−0.03	0.17
(15) Is employed	0.01	−0.04	−0.03	0.05	0.07	0.00	−0.16	0.32
(16) Has diabetes other than type 1	0.02	0.08	0.10	−0.03	−0.02	−0.02	−0.01	−0.30
(17) Diabetes duration (years)	0.07	0.08	0.05	0.05	0.00	0.01	0.17	−0.06
(18) Insulin duration (years)	0.08	0.08	0.05	0.06	0.00	0.00	0.17	−0.04
(19) Frequency will run high	−0.33	−0.32	−0.29	−0.30	−0.23	0.18	0.04	−0.12
(20) Has a comorbid condition	−0.09	−0.03	−0.02	−0.15	−0.11	0.10	0.13	−0.33
(21) Gold Score	−0.21	−0.17	−0.15	−0.20	−0.20	0.11	0.71	−0.16
(22) Uses insulin	−0.04	−0.07	−0.07	−0.03	0.00	0.09	0.00	0.10
(23) Uses CGM	−0.08	−0.13	−0.14	−0.03	−0.02	0.06	0.07	0.19

CGM, continuous glucose monitor

### Test–retest reliability

Three hundred and seventeen online participants consented to being re-contacted and the second survey was accessed 169 times. Of these participants, seven did not go on to complete the survey and 18 left the survey prior to the Hypo-RESOLVE QoL. One participant was excluded due to completion time (see above), leaving 143 for test–retest analysis. In a subsample that had a difference in scores on the VAS (4 weeks) within 1 SD of the mean (*n*=108; median 34 days between surveys), the estimated ICC was 0.87 (95% CI 0.80, 0.91), which represents good reliability [23].

## Discussion

Valid and reliable PROMs are indispensable tools for capturing patient-centred outcomes in clinical care and for assessing the effectiveness of healthcare interventions [27]. The Hypo-RESOLVE QoL is a novel 14-item measure, fit to an underlying theoretical framework of HRQoL, featuring a hierarchical three-domain structure of physical, social and psychological aspects and a superordinate HRQoL factor. The measure has good content validity and initial good supporting psychometric evidence, including structural validity, convergent validity, internal consistency and test–retest reliability.

### Strengths and weaknesses

The Hypo-RESOLVE QoL was developed in accordance with best practice, using qualitative and quantitative data synthesised from three studies with a combined sample of 1347 people with diabetes. The development process incorporated collaborative input throughout, including a high degree of patient engagement, clinician input and wider stakeholder engagement [28]. Iterative qualitative development work with individuals with diabetes, incorporating techniques designed to enhance trustworthiness, has produced a PROM with evident content validity. The absence of DIF by gender in the final PROM suggests the measure will function similarly across men and women. The resultant 14-item instrument was designed to help minimise respondent burden while balancing content coverage and psychometric performance, and facilitate subsequent work on producing a preference-based scoring system for use in cost-effectiveness trials for hypoglycaemia [12].

PROM development is iterative and, despite its evident strengths, there are some limitations of the Hypo-RESOLVE QoL that may be addressed in future studies. It would be inaccurate to claim that the reduced 14-item version of the Hypo-RESOLVE QoL would capture everything of interest to all individuals with diabetes. The comprehensiveness of the 14-item version cannot be determined without further qualitative research. A trade-off must be acknowledged between achieving adequate comprehensiveness and satisfactory psychometric performance. The importance of different items to people with diabetes who experience hypoglycaemia was incorporated into the item selection, based on information from underlying qualitative work and consultation with the PAC, to ensure important aspects were captured. The 14-item measure was approved by the PAC. However, it should be noted that the content validity of the final version was not assessed qualitatively, including its comprehensiveness. Further, some concepts identified as important to people with diabetes (e.g. sexual functioning) were not included in the final instrument due to unsatisfactory psychometric performance. Future work should assess the content validity of the PROM in independent samples, including the additive benefit of additional ‘bolt-on’ items to the core scale for use in particular groups.

While psychometric results in the development of the measure were generally positive, some minor issues should be acknowledged, such as the significant χ^2^ test when assessing goodness-of-fit. Nevertheless, it is well known that the χ^2^ test is of limited value in large samples, as it is almost always significant, even in the cases of trivial misfit [29, 30]. Further, only one item retained in the Hypo-RESOLVE QoL is positively framed. While efforts have been made to make this clear in the final questionnaire design, subsequent work should assess whether this affects responses.

The Hypo-RESOLVE QoL was developed predominantly in the UK and the sample was predominantly White. The PROM benefits from supplementary cognitive debriefing in another European country, Germany and a formal translatability assessment on the included content. Evidence from these sources is positive. Nevertheless, as is standard in the adaptation of PROMs to new countries and cultures [11], future research is required to ensure the PROM works well in other settings throughout Europe and internationally. This should include work with more ethnically diverse samples. Furthermore, future PROM development projects may consider involving linguistic experts earlier in item generation.

In relation to other studies and PROMs used in hypoglycaemia-related research, the Hypo-RESOLVE QoL benefits from assessing HRQoL as a holistic concept rather than having a narrow focus on specific QoL impacts, such as fear [31, 32]. We provide supporting evidence for the content and structural validity of the scale, which is absent in other hypoglycaemia-specific measures [4]. A weakness of the Hypo-RESOLVE QoL relative to other PROMs is a current lack of data on responsiveness to change in clinical trials, which is available for some diabetes-specific PROMs (e.g. [33]).

### Implications and future research

The Hypo-RESOLVE QoL can be used by clinicians and researchers in research and practice, including as a HRQoL outcome in clinical trials. A copy of the Hypo-RESOLVE QoL can be requested from the corresponding author. A subsequent preference-based scoring system will demonstrate utility in informing healthcare resource allocation decisions in hypoglycaemia, relevant to policymakers [12]. While a comprehensive body of development work is reported for the Hypo-RESOLVE QoL, including supporting psychometric evidence, studies in independent samples are required to further develop the evidence base for the measurement properties of the scale. This includes assessing the psychometric properties of the standalone 14-item version and, in particular, the responsiveness of the measure in clinical trials of intervention(s) for hypoglycaemia and its final comprehensiveness.

### Conclusion

The Hypo-RESOLVE QoL has been designed as a flagship output from the international Hypo-RESOLVE Consortium. The scale was developed in collaboration with, and for measuring HRQoL in, people with diabetes who experience hypoglycaemia, to demonstrate good content validity relative to existing hypoglycaemia-specific PROMs. Evidence from qualitative and quantitative studies suggest that the Hypo-RESOLVE QoL performs well in people with diabetes. The generation of additional data obtained from using the Hypo-RESOLVE QoL will be of value in furthering our understanding of the impacts of hypoglycaemia and how these may be best managed to optimise patient outcomes.

### Supplementary Information

Below is the link to the electronic supplementary material.

ESM Fig (PDF 131 KB)

## Data Availability

All data generated or analysed during this study are included in the published article and its online supplementary files (10.15131/shef.data.23295284.v2).

## Abbreviations

CFA: Confirmatory factor analysis
CFI: Comparative fit index
DIDP: DAWN2 Impact of Diabetes Profile
DIF: Differential item functioning
EQ-5D-5L: Generic measure of health-related QoL
HRQoL: Health-related QoL
HypoA-Q: Hypoglycaemia Awareness Questionnaire
Hypo-RESOLVE: Hypoglycaemia REdefining SOLutions for better liVEs
Hypo-RESOLVE QoL: Hypoglycaemia health-related QoL measure
ICC: Intraclass correlation coefficient
IRT: Item response theory
NHS: National Health Service
PAC: Patient advisory committee
PROM: Patient-reported outcome measure
QoL: Quality of life
RMSEA: Root mean square error of approximation
VAS: Visual analogue scale

## Authors’ relationships and activities

BEdG is a member of the Diabetologia Editorial Board. SH has received consulting fees from Eli Lilly (payment to International Hypoglycaemia Study Group), consulting fees from Zealand Pharma (payment to Institution), consulting fees from Zucara Pharma (payment to Institution), consulting fees from Novo Nordisk as part of Member of International Review Board (payment to Institution) and payment for expert testimony from patent case (personal payment); has participated on a Data Safety Monitoring Board or Advisory Board at Eli Lilly (payment to Institution) and is Chair of International Hypoglycaemia Study Group. JCo is an employee at Novo Nordisk. MR has stock or stock options at, and is an employee of, Eli Lilly and Company. M-AG is a shareholder and employee of Novo Nordisk. CJC holds stock or stock options at, and is an employee of Eli Lilly and Company. RJM has received payment or honoraria for lectures, presentations, speakers bureaus, manuscript writing or educational events from Sanofi. DR is a member of the Euroqol Group; SF-6Dv2 licensing royalties.
