# Supplementary material for: Development of a new health-related quality of life measure for people with diabetes who experience hypoglycaemia: the Hypo-RESOLVE QoL
Source: Diabetologia. 2024 May 22;67(8):1536–51. doi: 10.1007/s00125-024-06182-9 (PMC11343818; doi:10.1007/s00125-024-06182-9)
Supplement: Supplementary file 1 — ESM Fig (PDF 131 KB) [file 125_2024_6182_MOESM1_ESM.pdf]

ESM Figure 1. Illustration of differential item functioning by diabetes type in the final PROM.

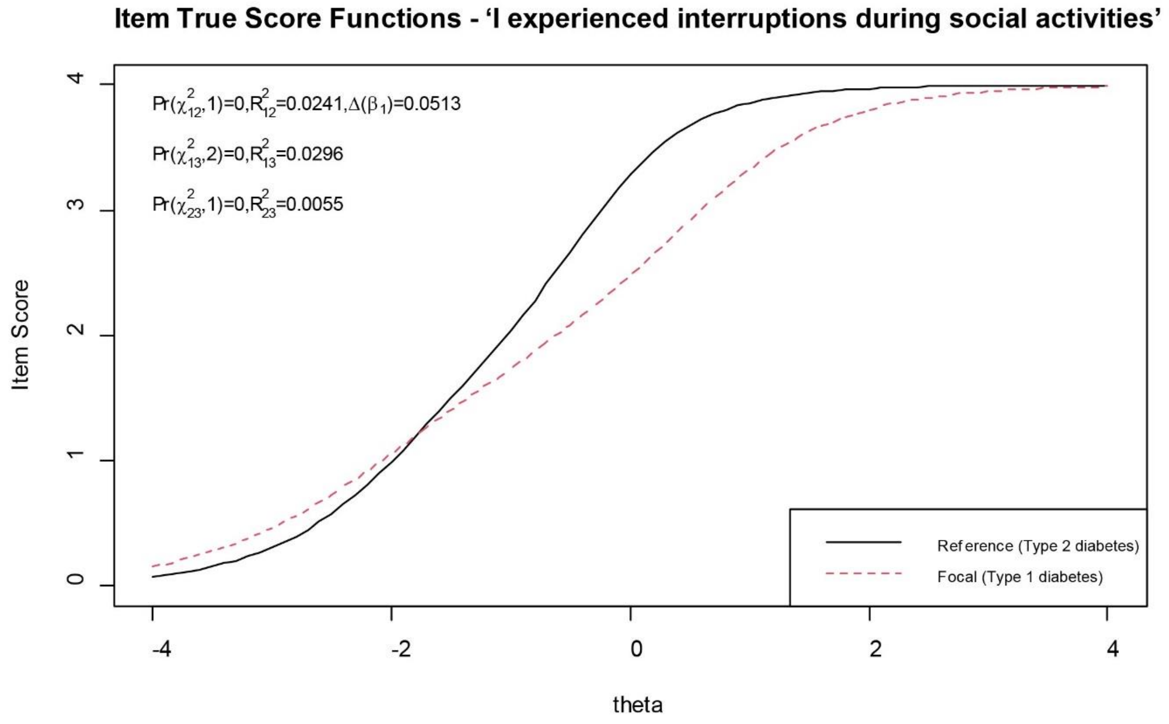

This figure was produced using the ‘lordif’ R Package (Choi et al., 2011). The item ‘I experienced interruptions during social activities’ demonstrated non-uniform differential item functioning (DIF) (McFadden R^2 = 0.0296, $p < .001$) between people living with Type 1 and Type 2 diabetes.

References

Choi SW, Gibbons LE, Crane PK. lordif: An R Package for Detecting Differential Item Functioning Using Iterative Hybrid Ordinal Logistic Regression/Item Response Theory and Monte Carlo Simulations. J Stat Softw. 2011;39(8):1-30. doi:10.18637/jss.v039.i08
